# Achieving high-performance parameters in NASICON-polymer composite electrolyte-based solid-state supercapacitors by interface modification†

**DOI:** 10.1039/d4ra08292c

**Published:** 2025-02-27

**Authors:** Neha, Anshuman Dalvi

**Affiliations:** a Department of Physics, BITS Pilani Pilani Campus RJ-333031 India adalvi@pilani.bits-pilani.ac.in

## Abstract

The present study reveals a strategy to enhance the performance of solid-state supercapacitors based on activated carbon electrodes and a Na_3_Zr_2_Si_2_PO_12_ (NZSP) dispersed fast ionic solid polymer electrolyte membrane. The electrode–electrolyte interface is optimized using a novel ‘solvent layer’ approach to enhance supercapacitor performance. By adding a small amount of acetonitrile organic solvent (a few μL cm^−2^) at the electrode–electrolyte interface and utilizing high surface area (1800 m^2^ g^−1^) activated carbon, significant improvements in specific capacitance, specific energy, specific power, and cycling stability are achieved. Device performance at various operating voltages and discharge currents reveals interesting results. A specific capacitance of approximately 260 F g^−1^ and a high specific power of 4780 W kg^−1^ is achieved at 3 V/5 mA. Moreover, after 10 000 galvanostatic charge–discharge cycles (1 V/1 mA), the supercapacitor exhibits ∼99% stable coulombic efficiency along with appreciably high capacitance retention (∼90%). A stack of five such cells can power an 8 V LED circuit for more than 30 minutes. Applying such a solvent layer enables effective use of the surface area of the activated carbon. Results suggest that solvent incorporation enables a local ‘gel-like’ layer formation that couples the electrode with a solid polymer electrolyte and facilitates faster charge movement across the electrode–electrolyte interface.

## Abstract 2

The present study reveals a strategy to enhance the performance of solid-state supercapacitors based on activated carbon electrodes and a Na_3_Zr_2_Si_2_PO_12_ (NZSP) dispersed fast ionic solid polymer electrolyte membrane.

## Graphical abstract

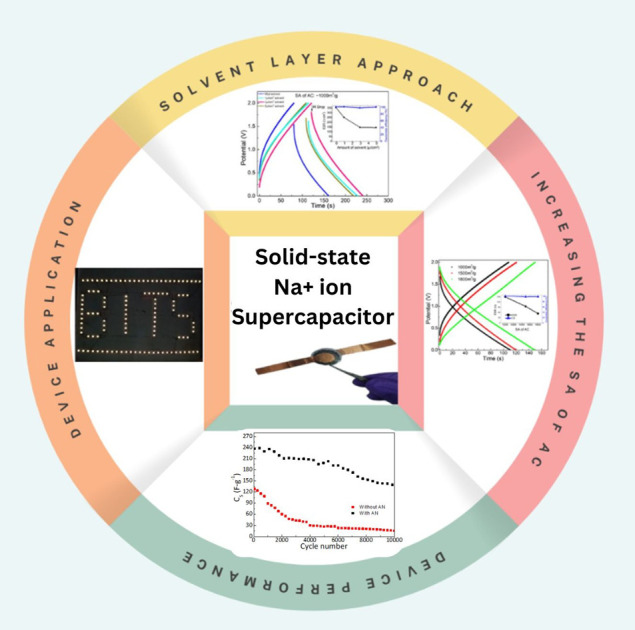

## 1. Introduction

The electric double layer, pseudo-solid-state supercapacitors, and new-generation Na^+^-ion batteries have drawn significant attention in recent years because of their potential applications in various devices, including electric vehicles and microgrids. Solid Na^+^-ion supercapacitors are particularly effective in scenarios where high power density, fast charging, long cycle life, and safety are essential. On the other hand, Na^+^-ion batteries are more suitable for applications that demand higher energy density and longer durations of energy storage. Supercapacitors are helpful in applications where faster charging–discharging is required. They also have longer cycle life than the batteries with minimal degradation. Supercapacitors excel over batteries in extreme temperature conditions. These advantages are crucial as they arise from the purely electrostatic nature of the charge storage process.

A significant emphasis has been placed on improving performance in terms of specific energy, power output, and long-cycling stability. In the past, various routes have been attempted to tailor their performance in order to achieve a balance between energy and power output. The use of high surface area activated carbon is an obvious choice.^1,2^ Other nanomaterials with high surface area, *e.g.*, graphene,^3^ also lead to improved performance. Thus, by modifying the structure of the electrodes, device performance can be notably customized. A high degree of porosity and pore connectivity in the electrode structure enables the electrolyte ions to reach the electrode surface and interior, leading to enhanced charge storage. For example, energy density reaches around ∼44 W h kg^−1^ when carbon fibers are used as electrodes.^1^ Further, using carbon nanotubes in the device provides capacitance in the range of 20–300 F g^−1^.^2^ Also, various approaches have been brought out for synthesizing high-performance supercapacitor electrodes using carbon hydroxide morphologies (rod, flower, and cube-shaped) to obtain high specific capacitance.^4^ Polymer salt composite has also been used as binder material to make electrodes more compatible, particularly with solid polymer electrolytes, to establish good contacts and utilize the maximum surface area of activated carbon.^5^

To design supercapacitors with wide temperature tolerance and electrochemical stability windows, a search for suitable electrolytes is also essential. Electrolyte conductivity influences capacitance as it provides pathways for ionic transport across the electrodes. The stability of an electrolyte is crucial when a supercapacitor works in a wide range of operating voltages. Further, the electric double-layer capacitance can be considerably impacted by the electrolyte viscosity and dielectric constant.^6^ The charge separation at the electrode–electrolyte interface can be tailored, the electrochemical stability (voltage) window can be increased and the total capacitance can be improved by selecting a suitable electrolyte with a high dielectric constant and low viscosity.^6^

Besides, the cell design has a major impact on the supercapacitor performance. Assembling the supercapacitors in 2032-button type cells, swage lock assembly, or in hot roll laminating geometry influences the device properties. The lamination method, which applies both temperature and pressure simultaneously to establish electrode-solid electrolyte contacts, has been shown to be an excellent way to improve interfacial charge movement.^7^

Interfacial contacts and electrode–electrolyte interfacial resistance also affect the device's performance. *In situ* polymerization processes based on oligomeric cyclotetrasiloxane and polyethylene glycol diglycidyl ether (PEGDE) have also been used to improve the interfacial resistance problem. With 10 wt% of CTS content, CSPE exhibits high ionic conductivity of 0.37 × 10^−3^ Ω^−1^ cm^−1^ at 28 °C. *In situ* polymerization has enhanced ionic transport at the electrode–electrolyte interface.^8^ Establishing interfacial contacts is quite challenging when solid electrolytes are attempted in supercapacitors. While liquid and gel electrolytes provide advantages *viz.* ease of ionic diffusion in the electrode pores and enhanced utilization of the electrode surface area, they also have certain disadvantages. The supercapacitors fail to withstand wide temperature variations and external pressures. Further, device miniaturization is tricky when liquid or gel electrolytes are used. Therefore, exploring solid–solid electrolytes is inevitable in supercapacitors.^9^

Ionic liquid-added NASICON-type^10^ and perovskite-type^11^ Li^+^-ion conductors have shown remarkable conductivity enhancement and are effectively used as supercapacitor electrolytes.^12^ The liquid-free, solid-state supercapacitors (SSCs) are relatively less explored. Solid-state electrolytes, *viz.*, NASICONS, garnets, and perovskites-based composite polymers, have been quite successfully applied in solid-state batteries.^5,9,13,14^ Developing solid-state supercapacitors (SSCs) along with SSBs is an important approach as their combination can lead to new generation smart energy storage systems. Importantly, similar to solid-state batteries, there is a need to develop ‘liquid-free’ SSCs utilizing fast ionic solid electrolyte. However, since supercapacitance is an interfacial phenomenon, such usage of solid electrolytes is only possible provided the interface is notably improved. Direct application of ceramic fast ionic systems, *e.g.* garnet or, NASICON type fast ionic conductors is rather restricted due to rough interface. However, a desired interface can be achieved using a highly conductive (≥10^−4^ Ω^−1^ cm^−1^) flexible composite solid polymer electrolyte membrane.^5,14^

In our recent studies, NASICON-dispersed PEO–NaCF_3_SO_3_ membranes were developed by our group with high ionic conductivity and found to be potential solid electrolytes for batteries and supercapacitors.^5,13^ We realized that prior to application, it is also essential to address the interface between the electrode and electrolyte to ensure stability and successful long charge–discharge cycling. Improved solid–solid interfacial contacts can enhance the effective utilization of the surface area of the activated carbon, and high power output and long, stable cycling performance can be realized.

The current study, therefore, addresses a method to improve the solid–solid interface by introducing a thin layer of organic solvent.^15^ Study reveals that the supercapacitor performance parameters can be further improved once the interface is well connected by choosing high surface area (SA) activated carbon. Thus, the solvent layer facilitates the effective use of a surface area of activated carbon.

## 2. Experimental

### 2.1. NASICON (NZSP) synthesis

**Fig. 1:**
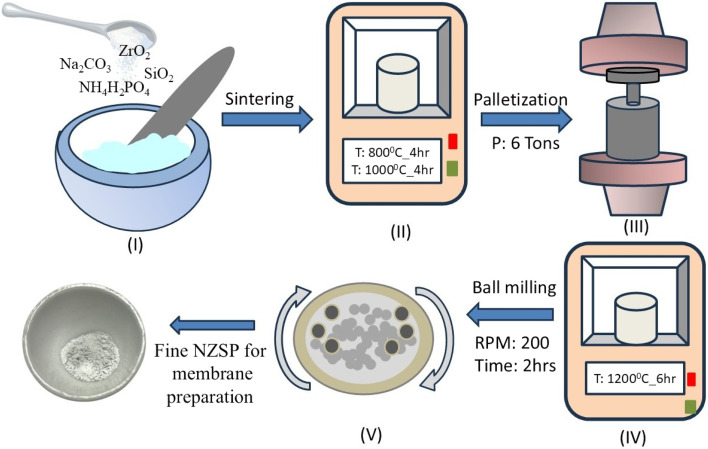
Schematic depicting a five-step synthesis process of Na_3_Zr_2_Si_2_PO_12_ (NZSP).

The NASICON structured NZSP (Na_3_Zr_2_Si_2_PO_12_) is prepared *via* the solid-state reaction route^5,16,17^ depicted in Fig. 1. Initially, Na_2_CO_3_, ZrO_2_, SiO_2_, and NH_4_H_2_PO_4_ were weighed according to their molar ratios and properly mixed using a pestle and mortar. This mixture was then transferred to an alumina crucible and placed in a muffle furnace, where it underwent step heating: 4 hours at 800 °C followed by 4 hours at 1000 °C. After allowing the furnace to cool naturally, the powder was used to prepare pallets (thickness 2–3 mm, diameter 10 mm) using 5 ton pressure. Subsequently, pellets were sintered at 1200 °C for 6 hours. Finally, the furnace-cooled pallets were taken out for X-ray diffraction and conductivity measurements.

To prepare the CSPE membrane, the pellets were ground into a powder using mechanical milling in a planetary ball mill (Fritsch P-6) for 2 hours in an 80 mL Zirconia pot, maintaining a fixed ball-to-sample mass ratio of 5 : 1. Thus, the fine-milled NZSP was used for the membrane preparation.

### 2.2. Polymer–NZSP composite electrolyte and device fabrication

**Fig. 2:**
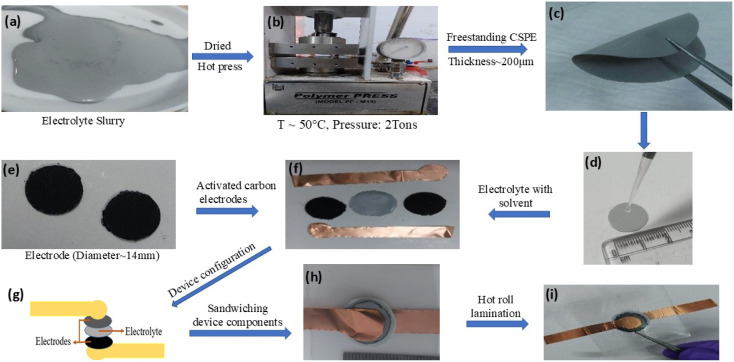
Preparation steps for the composite solid polymer electrolyte (CSPE) membrane and the supercapacitor fabrication: (a) composite electrolyte slurry (b) hot press for CSPE membrane preparation, (c) flexible CSPE membrane, (d) solvent addition at the CSPE surface, (e and f) supercapacitor components, (g and h) schematic diagram and the supercapacitor in sandwich geometry, and (i) final laminated supercapacitor.

The synthesis process employed for polymer–salt–NZSP composite membrane preparation is shown in Fig. 2a–g. Adopting the solution casting route, the salt (NaCF_3_SO_3_) and NZSP crystallites were disseminated uniformly in the host polymer (PEO: MW 300 000 g mol^−1^) matrix (composition: 10NaCF_3_SO_3_–90(0.4PEO–0.6NZSP)). The solution was continuously stirred for 9 h, until the formation of a thick slurry that was further dried at room temperature for 12 hours. The dried slurry was subsequently hot-pressed at 2 tons at 60 °C to produce a ∼0.2 mm composite membrane (Fig. 2c).^5^

The supercapacitor electrodes were prepared by blending high surface area activated carbon (AC), acetylene black, and a salt-added polymer (0.8PEO–0.2NaCF_3_SO_3_) in a weight ratio of 70 : 10 : 20, respectively, in DI water medium. The slurry, thus obtained after 24 h stirring, was applied on a 0.5 mm thick graphite sheet by standard doctor blade technique and kept for 12 h drying in a vacuum oven at 45 °C. Finally, the coated sheets were cut into 15 mm diameter discs (mass loading ∼0.6 m^2^ g^−1^) and used for the supercapacitor fabrication (Fig. 2e). High mass loading is avoided to prevent a resistive solid electrode–electrolyte interface.

Solid-state supercapacitors (SSCs) were fabricated by using a CSPE membrane with ∼3–5 μL cm^−2^ of acetonitrile applied on the surface of the CSPE membrane (Fig. 2d) and the electrodes in configuration graphite|AC|electrolyte|AC|graphite (Fig. 2g) using a hot roll laminator operated at ≥90 °C (Fig. 2h and i).

### 2.3. Device characterization

The CSPE electrolyte films and electrode–electrolyte interface before and after the application of AN were analyzed by field emission scanning electron microscopy (FEI-Apreo-S, accelerating voltage range: 200 V–30 kV) and Raman Spectroscopy (Labram HR Evolution, 532 nm Nd–YAG laser 100 mW). X-ray diffraction measurements were carried out by using a Rigaku Smartlab HT-X-ray diffractometer with CuK_α_ radiation (*λ* = 1.54 Å). The tensile test is performed using Instron_34SC-5 single model universal testing machine of dimension 35′′ : 46′′ : 62′′. The displacement rate was set to 1 mm min^−1^ to observe the slightest change in membrane morphology. The electrochemical properties of the SSCs were evaluated using a Gamry Interface 1010E electrochemical workstation. Galvanostatic charge–discharge cycles were obtained at different current and voltage levels to study the properties of the SSCs.

Supercapacitor performance was evaluated using the following relations:^18^

(i). Using cyclic voltammetry (CV) curves, the following parameters could be obtained:i
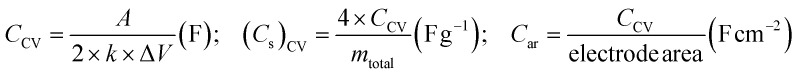
where *C*_CV_ is the capacitance as obtained from the CV curve, *C*_ar_ is the areal capacitance of the device. Further, (*C*_s_)_CV_ is the specific capacitance value per electrode. Here, *A* represents the area under the CV curve, *k* is the scan rate, and Δ*V* is the voltage window.

(ii). From galvanostatic charging–discharging (GCD) cycles using discharge current (*I*) and discharge time (Δ*t*), various performance parameters were obtained.ii
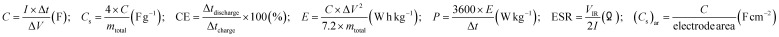
where *C* represents the capacitance as obtained from GCD cycles, *C*_s_ is the specific capacitance per electrode, CE is the coulombic efficiency, *E* and *P* are the specific energy and specific power corresponding to the full device, ESR the equivalent series resistance, and Δ*V* the discharge voltage excluding the IR-drop. Here, *m*_total_ (kg) is the total mass of active material on both electrodes.

The steady-state electrical conductivity of the NZSP pallet and CSPE membrane was measured using the Hioki IM3570 impedance analyzer^5^ in a wide frequency range of 4 Hz–5 MHz. For the NZSP pallet conductivity, graphite paint was used for electrical contacts. The CSPE membrane was sandwiched between stainless steel electrodes prior to measurements.

## 3. Results and discussion

### 3.1. NZSP: structure and conductivity

X-ray diffraction patterns of the NZSP pallet are shown in Fig. 1a. The peaks indeed confirm the formation of Na_3_Zr_2_Si_2_PO_12_ (NZSP). All the peaks match well with the JCPDS data except for some tiny peaks corresponding to ZrO_2_ impurity. Few peaks are slightly shifted indicating lattice strain possibly due to impurities.

**Fig. 3:**
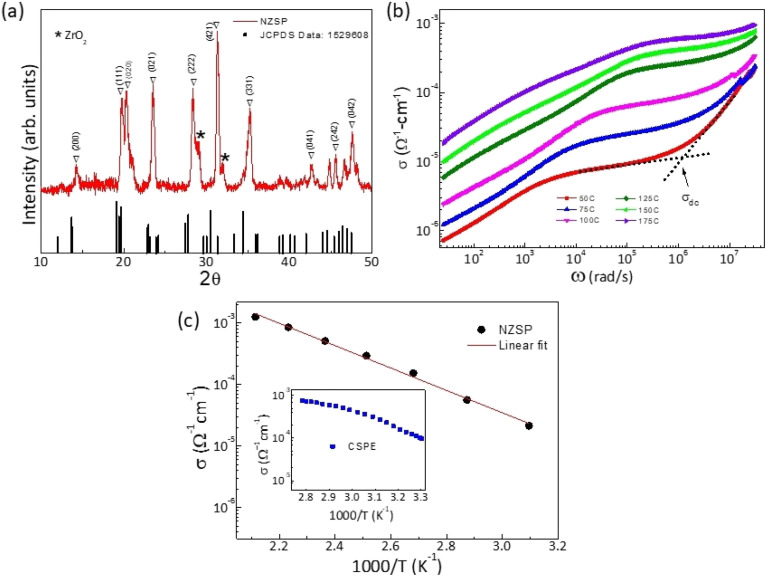
(a) X-ray diffraction of the as-prepared NZSP along with JCPDS data (card number: 1529608); (b) conductivity *vs.* frequency plot of pristine NZSP pallet at different temperatures; (c) temperature dependence of conductivity for pristine NZSP pallet and CSPE membrane in the inset.

The frequency dependence of electrical conductivity is obtained for the NZSP pallet (Fig. 3b). The conductivity exhibits dc to dispersion region at higher frequencies and a pleatue at mid frequencies followed by gradual fall at lower frequencies. This is a typical behaviour confirming the predominantly ionic nature of NZSP. The DC conductivity was obtained from the plateau region. Conductivity–temperature cycles for pristine NZSP and its composite-polymer electrolyte with polymer (10NaCF_3_SO_3_–90(0.6NZSP–0.4PEO)) are shown in Fig. 3c and its inset. The conductivity, though, depends upon sintering temperature and time. The highest conductivity value of 1.8 × 10^−5^ Ω^−1^ cm^−1^ is observed at 50 °C for the NZSP pallet, which matches quite well with the previous studies.^19^ On the other hand, the NZSP dispersed CSPE membrane exhibits much higher conductivity (2.7 × 10^−4^ Ω^−1^ cm^−1^ at 40 °C) and is found suitable for electrolytic applications. The CSPE composition contains 54 wt% of NZSP. On further increasing the NZSP content, ceramic content becomes quite significant in the matrix, thus leading to poor interfacial contacts. Therefore, more NZSP reinforcement was avoided, and the composition 10NaCF_3_SO_3_–90(0.60NZSP–0.40PEO), abbreviated as 60NZSP, was used for the electrolytic applications. Additional characterization of other NZSP-content polymer membranes is reported earlier.^5^

### 3.2. Acetonitrile at the electrode–electrolyte interface

**Fig. 4:**
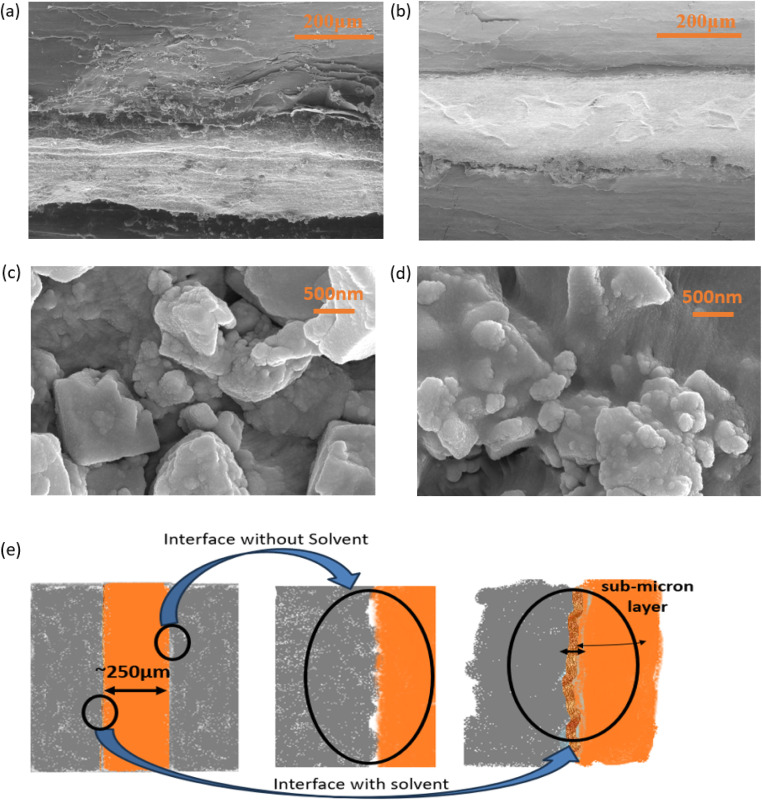
(a) SEM image of device cross-section showing the effect of acetonitrile application on the interface. (a) Without AN and (b) with AN. Effective contact area apparently improves on AN application. FESEM images of composite solid polymer electrolyte (CSPE) membrane surface (c) before and (d) after applying AN. (e) Schematic representation of the effect of AN addition at the interface.

Fig. 4a and b show scanning electron microscopy images of the device's cross-sectional area before and after acetonitrile (AN) application at the electrolyte surface. AN between electrode and electrolyte improves the interface and establishes smooth contact.

Fig. 4c and d show FESEM images of the surface of CSPE with and without the presence of AN. When acetonitrile is applied, the surface appears smoother, and the polymer spreads evenly. The presence of AN also eliminates any pores or cavities on the surface.

Finally, Fig. 4e shows pictorially how the interface gets modified due to AN presence.

The effect of AN application on the CSPE surface is further investigated. After the solvent is added to the CSPE surface, an increase in elongation is observed due to softening. Further, the yield strength gradually decreases as the amount of acetonitrile (AN) increases. These results indicate that acetonitrile diffuses into the polymer matrix, altering its mechanical properties. The diffusion is, however, limited as the AN is added in a small amount.

**Fig. 5:**
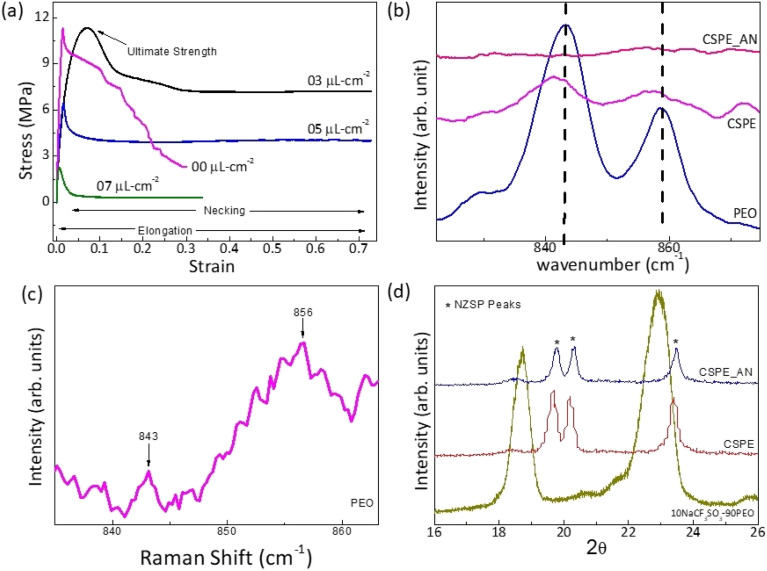
(a) Stress–strain curve of CSPE membrane with different amounts of acetonitrile content. (b) Raman spectra for the surface of polymer electrolyte before and after applying AN on the surface, (c) Raman spectra of CSPE after AN application on an extended scale (d) X-ray diffraction of CSPE membrane with and without the addition of solvent membrane.

In order to further understand the effect of AN addition on the local structure at the surface, Raman spectra were collected at room temperature for the CSPE with and without the addition of AN. As shown in Fig. 5b, the attributary peaks are compared to pristine PEO film prepared using the same process. This spectra range is chosen as there is no overlap of PEO absorption peaks with those of NZSP, and Raman shift corresponds only to PEO Raman active modes. For the untreated PEO membrane surface, the Raman spectra (Fig. 5b) show peaks at 843 cm^−1^ (the C–O stretching vibration + the CH_2_ rocking vibration) and 856 cm^−1^ (the C–C stretching vibration + the C–O stretching vibration + the CH_2_ rocking vibration). Further, the signal at 843 cm^−1^ is assigned to the vibrations of molecules in the helical conformation in the monoclinic crystalline phase. As expected, the untreated PEO film exhibits characteristic narrow Raman bands, attributed to the ordered arrangement of chains^20,21^ as reported earlier.

The CSPE membrane has a relatively smaller content (36 wt%) of PEO. Further, the salt and NZSP addition leads to a decrease in the crystallinity.^5,22,23^ Thus, the peak height and area are reduced substantially. Further, a broadening witnessed in the Raman band may be attributed to the disorder in the polymer chains.

Apparently, the Raman peaks are of substantially low intensity (Fig. 5c) for the CSPE after adding AN to the surface. The low intensity possibly suggests a decrease in the sample concentration and, thus, the number of scattering centers. Such a nature of Raman peak is indicative of gel formation of the surface. It may be suggested that the AN diffused regions behave like a gel, a mixture of solvent and polymer. This ‘localized gel layer’ enhances interfacial contacts when the surface is in contact with the electrode.

XRD patterns of the CSPE surface before and after the addition of AN are shown in Fig. 5d. The peaks corresponding to the pristine PEO membrane are also shown for comparison. Apparently, PEO peaks completely disappear in the CSPE membrane. Additionally, upon adding AN, the XRD patterns remain unchanged, and the peaks related to NZSP stay intact.

**Fig. 6:**
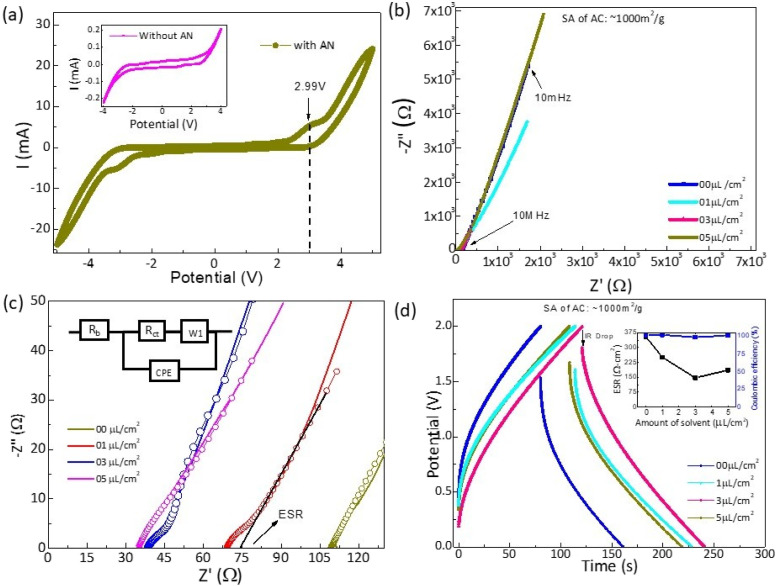
(a) Cyclic voltammetry (100 mV s^−1^) for CSPE membrane with and without AN addition. (b) Nyquist plot of the device, (c) Nyquist plots on an extended scale with corresponding equivalent circuit; (d) GCD plots of devices fabricated with varying amounts of acetonitrile at the electrode–electrolyte interface. (Inset) ESR and coulombic efficiency *vs.* amount of AN. These results correspond to supercapacitors with activated carbon electrodes of SA ∼1000 m^2^ g^−1^.

At the outset, the effect of AN addition on the surface of CSPE was assessed on the electrochemical stability window of the electrolyte. Fig. 6a shows cyclic voltammetry plots of CSPE and AN-added CSPE membrane when sandwiched in between stainless steel electrodes. After the addition of AN, no significant change is observed in the potential window except a small kink around 3 V (as shown by the arrow). One may, however, notice that the peak current increases notably for AN-added CSPE. This rise may be attributed to enhanced mobile ion activity due to gel layer formation.

Fig. 6b shows the Nyquist plot (1 mHz–1 MHz) for the supercapacitor cell with the variation of AN content at the interface. The low-frequency inlined line is relatively steeper for ∼3 and 5 μL cm^−2^ content of AN, suggesting pure capacitor behavior. Fig. 6c shows the same Nyquist plots on an extended scale, particularly at higher frequencies. The equivalent circuit shown in the figure fits well with the Nyquist plots. The parameters like charge transfer resistance *R*_ct_, bulk resistance (*R*_b_), and equivalent series resistance (ESR) are presented in Table S1 (ESI).† ESR reduces substantially as a result of AN addition, resulting in an improved interface.

The GCD cycles for SSC cells with and without AN are shown in Fig. 6d. Interestingly, due to AN addition, discharge time significantly increases. The IR drop (that leads to ESR, *V*_IR_/2*I*) is significantly reduced and found to be comparable to many gel-based SSCs reported earlier.^24,25^

### 3.3. Effect of surface area enhancement

**Fig. 7:**
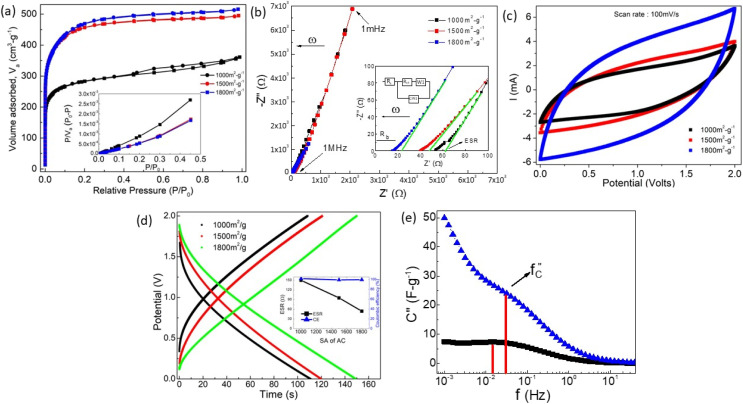
(a). Nitrogen adsorption–desorption isotherm curves for different surface area activated carbon (AC) electrodes (parameters in ESI Table S2†); (b) device Nyquist (EIS) plot, on the extended scale in the inset with equivalent circuit (c) cyclic voltammetry, (d) galvanostatic charge–discharge cycles ((Inset) ESR and coulombic efficiency as a function of surface area of AC) for different surface area activated carbon (∼1000 m^2^ g^−1^, ∼1500 m^2^ g^−1^ and ∼1800 m^2^ g^−1^) electrodes. (e) Imaginary capacitance (*C*′′) *vs.* frequency.

After optimizing the AN content at the interface (to ∼3 μL cm^−2^), the surface area of the AC was gradually increased to examine the scope of further improvement in the performance parameters. Prior to application, the porous structure and surface area of AC were analyzed using Nitrogen adsorption–desorption. The measured Brunauer–Emmett–Teller (BET) surface area (Fig. 7a) of AC materials was found to be ∼1000 m^2^ g^−1^, ∼1500 m^2^ g^−1^ and ∼1800 m^2^ g^−1^. A Type IV hysteresis is featured in the isotherm corresponding to the 1000 m^2^ per g SA sample. The hysteresis is attributed to the trapping of N_2_ within the mesopores structure. Due to their small pore size, such an AC material is more prone to faradaic charge storage mechanisms.^26^ Further high SA samples, typical type-1 isotherm is witnessed. The presence of micropores (≥50 nm) is evident due to the presence of near-verticle adsorption isotherm at a relatively low pressure <0.2. Such adsorbents are expected to enhance the electrochemically active surface area to attain high capacitance.^27^

From the Nyquist plot (Fig. 7b), it is evident that ESR decreases with an increase in the surface area of activated carbon. It is evident from the CV curve (Fig. 7c) that the area under the curve and, hence, charge storage ability increases, and the mobile ion uses the SA effectively.

The capacitance value obtained by the CV curve (Fig. 7c) is seen to be highest for a cell with ∼1800 m^2^ g^−1^ SA (*C*_s_ ∼ 100 F g^−1^ or *C*_ar_ ∼ 20 mF cm^−2^) against a cell with ∼1000 m^2^ g^−1^ (*C*_s_ ∼ 43 F g^−1^ or *C*_ar_ ∼ 9 mF cm^−2^). A similar trend is also seen in the GCD plot where the specific capacitance value rises from ∼215 F g^−1^ (*C*_ar_ ∼ 84 mF cm^−2^) to ∼263 F g^−1^ (*C*_ar_ ∼ 102 mF cm^−2^) with surface area increment (Fig. 7d). The ESR values have also significantly decreased, depicting improved interfacial contacts.

The 
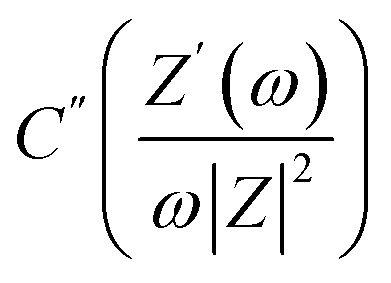
*i.e.*, the imaginary part of the capacitance, provides an important understanding of the charge storage at the interface.^28^ The graph of *C*′′ *versus f* exhibits a relaxation and the peak corresponds to a relaxation frequency (*f*_C_) for charge accumulation at the pores of the electrode. A shift of *f*_C_ to higher values suggests that mobile ions require relatively less time to reach the electrode pores. As apparent in Fig. 7e, the SSC with 1800 m^2^ per g SA shows a relaxation peak at 27 mHz corresponding to the lowest time of 37 s in comparison to the relatively low frequency of 14 mHz (∼72 s) in the case of 1000 m^2^ per g SA activated carbon electrodes.

In all three cells with different SA, the coulombic efficiency maintains a constant value (≥99%) at 2 V/1 mA. This readily suggests that the time required for the ions to reach the AC surface/pores and to go back to the bulk of the electrolyte is almost the same. These reversible smooth pathway for mobile ions and their formation to attributed to (i) the presence of polymer in electrodes^4^ and (ii) AN presence at the interface that leads to better compatibility.

### 3.4. Performance of supercapacitors with optimized AN content

The so-optimized supercapacitor with ∼1800 m^2^ per g SA and a solvent (AN) layer of ∼3–5 μL cm^−2^ at the interface (both sides) was thus used for further long cycling studies.

**Fig. 8:**
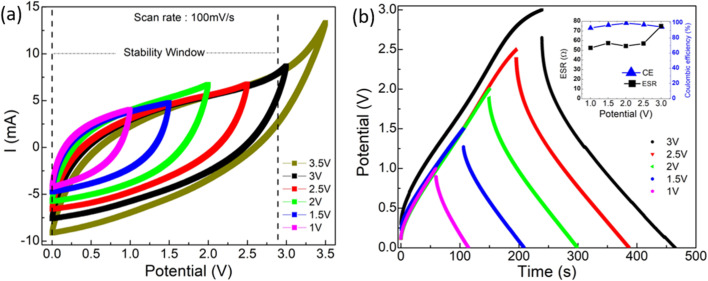
(a) CV (b) GCD curves for optimized supercapacitor (SA ∼ 1800 m^2^ g^−1^ and AN ∼ 3 μL cm^−2^) at different operating voltages.

Fig. 8a depicts the stability of the device up to a cutoff voltage of 3.5 V. The CV curves exhibit no notable variation from a box-like nature up to ∼2.75 V. Above 3 V, a slight deviation is observed. At 3.5 V a capacitance value of ∼22 mF cm^−2^ (∼116 F g^−1^) is obtained. On the other hand, the GCD curves (Fig. 8b) exhibit a triangular shape at low operating voltages. Such a variation suggests a possible deviation from electric double-layer type behavior above 2.5 V. Inset (Fig. 8b) shows that the coulombic efficiency remains >90% up to 3 V, and the ESR increases for higher operating voltages. Nevertheless, a high capacitance value (*C*_s_ ∼ 264 F g^−1^ or *C*_ar_ ∼ 102 F cm^−2^) is obtained for 2 V compared to ∼200 F g^−1^ at 2 V operating voltage obtained for 1000 m^2^ per g SA electrode. These results are consistent with CV and suggest that a ∼2 V operating voltage is safe for the SSCs.

**Fig. 9:**
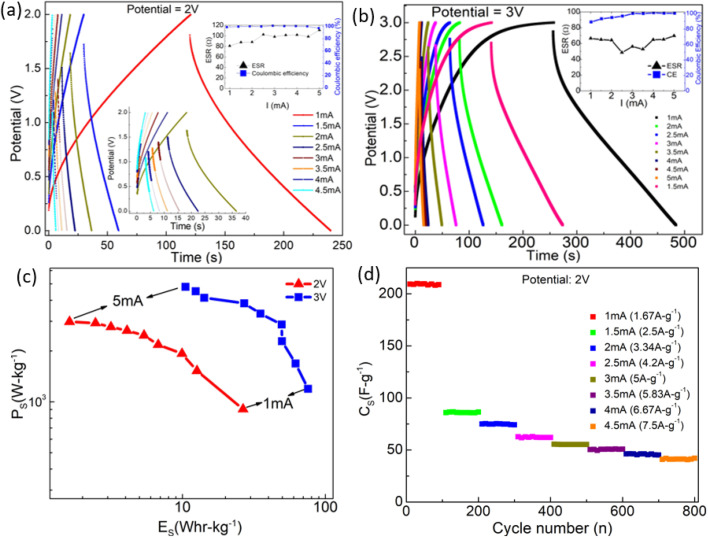
GCD plots for different currents at operating voltages of (a) 2 V and (b) 3 V, (c) Ragone plot for supercapacitors operating at 2 V and 3 V. (d) *C*_s_*vs.* cycle number for different discharge currents at 2 V.

The performance of the SSC is also tested at different discharge current values (Fig. 9). The GCD cycles show linear discharge behavior. The ESR, however, increases for higher discharge currents, that may be attributed to polarization at the interface, quickly builds up due to fast charging. Typically, for 5 mA, ESR is ∼100 Ω, whereas for 1 mA, it reduces to ∼80 Ω. Thus, for higher discharge current (5 mA), the ESR is still comparable to the earlier reported values for the gel-based supercapacitors.^29–31^Fig. 9a (inset) shows that the CE exhibits almost a constant value close to ≥99% with various discharge currents.

Fig. 9b depicts GCD cycles (3 V) at various discharge currents. The CE improves dramatically, and ESR remains stable (<60–70 Ω) for higher currents. Thus, the performance is satisfactory even at higher operating potentials (3 V).

The Ragone plot at two different operating voltages is shown in Fig. 9c. A maximum power density of ∼4780 W kg^−1^ is obtained at a discharge current of ∼8.34 A g^−1^ (5 mA) at 3 V. However, for such currents, the charge–discharge time reduces. A capacitance value of ∼265 F g^−1^ is attained with ∼1.67 A g^−1^ (2 mA) discharge current for a potential of 3 V.

At an operating voltage of 2 V, Fig. 9d shows the variation of *C*_s_ with cycle number. After every 100 cycles, the capacitor is discharged with a slightly higher current (0.5 mA). The *C*_s_ value is almost constant as long as the discharge current is kept constant. It, however, drops to a relatively lower value as the discharge current increases. The *C*_s_ value varies from ∼40 F g^−1^ to 220 F g^−1^ when the discharge current changes from 1 mA to 5 mA. Interestingly, the fall in the *C*_s_ value with discharge current variation is initially substantial. For subsequent cycles, this change is less prominent. Such behavior may be attributed to local polarization during the initial cycles. In the later cycle, a change in *C*_s_ is less significant, possibly due to the formation of pathways for mobile ions to reach the electrode pores. Nevertheless, up to 4.5 mA, *C*_s_ remains quite stable with currents.

### 3.5. Mechanism of charge storage

The mechanism of capacitor formation is also assessed by evaluating the separate contribution of pseudo and electric double-layer behavior of the capacitance. This is obtained from CV analysis using Dunn's method.^32^ There are various reports on how the current relates to the potential scan rate in a typical CV.^33^ In a broader sense, the relationship between peak current and scan rate is given by:^26^

iii *i*_p_ = *av*^*b*^

where *a* and *b* are constants whose values decide the nature of the charge storage process. When the current is controlled by capacitive processes, the *b* value approaches unity, and when it is controlled by diffusion-controlled processes, it reaches a value of 0.5.^34^ For more complicated systems that involve various charge storage processes, the peak current is a combination of the currents due to capacitive (electrical double layer formation at the interface) and diffusion-controlled processes when the ion diffuses into an electrode for a charge transfer as per the following relation:

iv *i*_p_ = *k*_1_*v* + *k*_2_*v*^1/2^

where *k*_1_ and *k*_2_ are the constants such that in *i*(*v*)/*v*^1/2^*vs. v* plot gives a straight line with *k*_1_ as slope and *k*_2_ as intercept. Due to factors including non-porosity of the surface electrode and varied kinetic behavior at different scan rates, this relationship is not necessarily linear in the required scan rate range.^33^

**Fig. 10:**
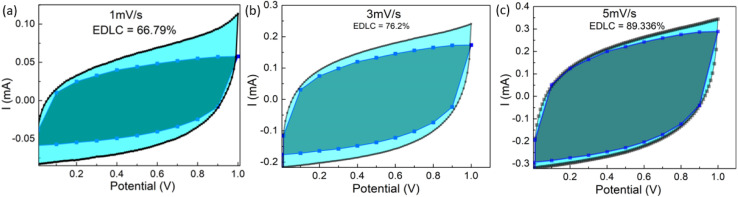
For the cells with SA ∼1800 m^2^ g^−1^ and AN ∼ 3 μL cm^−2^, the plots depicting EDLC contribution in capacitance for (a) 1 mV s^−1^, (b) 3 mV s^−1^, and (c) 5 mV s^−1^. The shaded region within the actual CV represents the electric double-layer contribution in total capacitance.

The contribution of pseudo and EDLC to charge storage in supercapacitors is assessed and shown in Fig. 10. It is essential to perform cyclic voltammetry scans at low rates (*e.g.* 1–10 mV s^−1^) in order to get meaningful information.^34^

CV curve was obtained at various scan rates (1, 3, 5, 7 and 9 mV s^−1^) to estimate EDLC/pseudo contributions in the charge storage mechanism. Fixing the potential, current values corresponding to different scan rates were recorded and used for the analysis. The interval between the two potentials was kept minimum (0.1 V) in order to obtain optimum results. Finally, a plot between *i*/*v*^1/2^*vs. v* for a fixed potential was obtained that exhibited a straight line with *k*_1_ (slope) and *k*_2_ (intercept). Substituting the values of constants *k*_1_ and *k*_2_ at different scan rates in eqn (iv) gives capacitive current. The process was repeated for different potential values. Finally, a current *vs.* potential plot for a given scan rate was obtained.

As shown in Fig. 10, increasing the scan rate causes a notable decrement in the pseudo capacitance of the device. For cells with minimum surface area (∼1000 m^2^ g^−1^ (Inset: ESR variation with cycle number)) of active material at the electrode, as the scan rate is increased, the EDLC contribution changes from ∼37% (1 mV s^−1^) to ∼73% (5 mV s^−1^), respectively. However, for the cells with higher surface area (∼1800 m^2^ g^−1^), EDLC contribution appears predominant, and it varies from ∼66% to ∼89%. As the surface area of the active electrode material increases it leads to a favorable situation for ion adsorption. This, in turn, leads to a higher charge density, resulting in a considerable increase in the supercapacitor performance parameters. The higher SA leads to more available sites, allowing ions to readily adsorb onto the electrode and causing a denser accumulation of charges. This translates directly to a higher capacitance. Moreover, the shorter diffusion paths within the electrode pores allow ions to reach the surface faster, facilitating quicker charge and discharge cycles.^34^ Thus, as the surface area of active material increases, the likelihood of a surface reaction decreases, making it a better EDLC.^35^

**Fig. 11:**
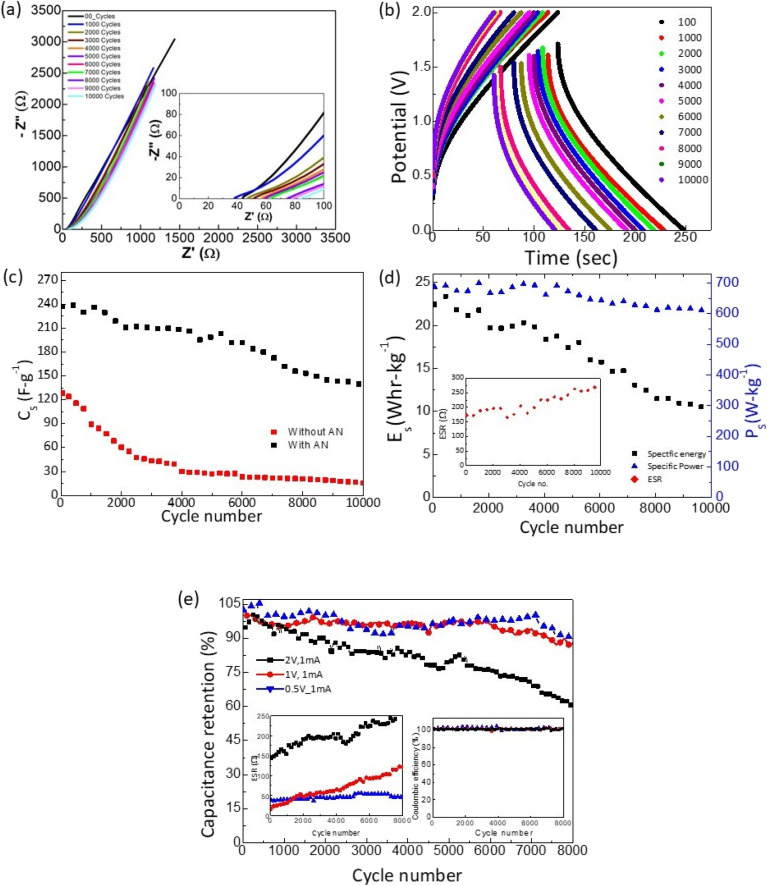
For supercapacitor with ∼3 μL per cm^2^ AN at interface and 1800 m^2^ per g SA: (a) device Nyquist (EIS) plots ((Inset) same Nyquist plot on extended scale at higher frequency), (b) galvanostatic charge–discharge cycles (2 V/1 mA), (c) *C*_s_ as a function of cycle number for devices with and without AN layer, (d) specific energy and Specific power as a function of cycle number ((Inset) ESR variation with cycle number). (e) Capacitance retention (%) *versus* cycle number at different operating voltages. Inset of (e) shows the ESR and CE with cycling at different operating voltages.

The SSCs were finally tested for long cycling. As shown in Fig. 11a, the Nyquist plots obtained after every 1000 GCD cycles exhibit an almost similar nature. The intercept of the inclined line with the *Z*′ axis slightly shifts to a higher value. Accordingly, the corresponding ESR shows a subtle rise. Further, the GCD cycles also suggest a similar nature with a slight increase in voltage drop during discharge. The specific capacitance is plotted as a function of cycle number for the device with and without an AN layer, as shown in Fig. 11c. The *C*_s_ value decreases initially and stabilizes substantially to a value of ∼150 F g^−1^. The capacitance retention of ∼60% is seen for the SSCs. Throughout the cycles, the coulombic efficiency remains almost stable at ≥99%. On the other hand, cycles without AN exhibit much lower values. For this, the *C*_s_ falls initially rapidly and stabilizes to a much lower value in subsequent cycles.

The almost constant nature of specific power and energy *vs.* cycle number (Fig. 11d) throughout the experiment is appreciable. Specific power drops to 610 W kg^−1^ from 700 W kg^−1^, depicting ∼90% retention even after ∼10 000 cycles.

Fig. 11e shows the capacitance retention (%) *vs.* cycle number at different operating voltages (0.5–2 V) for the same discharge current (1 mA). As apparent in the inset, for all the voltages coulombic efficiency is maintained to a constant value of ∼99% throughout cycles. Interestingly, the ESR for operating voltages of 0.5 and 1 V shows a substantial decrease. For 0.5 V it is maintained below 50 Ω even after ∼8000 cycles, showing less device deterioration at low operating voltages. After ∼8000 cycles for low operating voltages, the capacitance retained up to ∼90% of the initial value. Optimizing discharge current and operating potential can significantly tailor capacity fading and ESR. Apparently, the cell performance is more stable at low voltages.

**Fig. 12:**
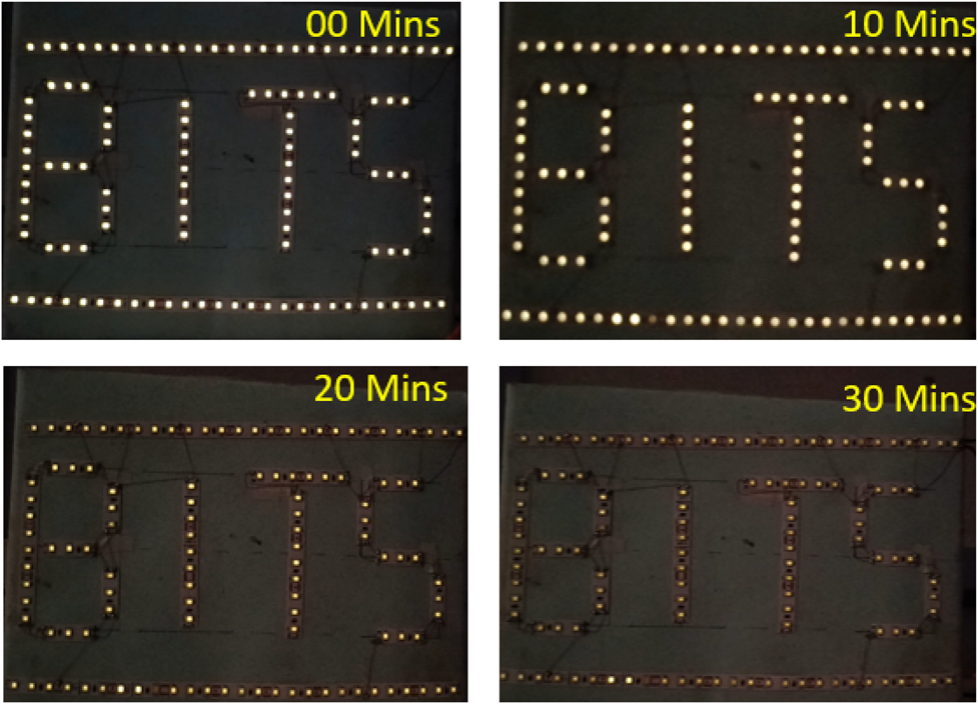
Five-cells linked in series make a glowing LED circuit (8 V).

To demonstrate the practical applicability of the SSCs, the series combination of five optimized cells has been used to glow a white LED (8 V) circuit as seen in Fig. 12. During direct discharge, the LED could light up for more than 30 minutes at a temperature of about 25 °C.

Above results clearly demonstrate that AN is vital at the interface. The improved interface not only maximizes the utilization of the AC surface area but also ensures smooth charge transfer during cell operation. It is evident that such an incorporation creates a thin ‘local gel-like layer’ at the interface that allows the electrolyte ions to reach to the electrode pores. Further, it is also evident that since the amount AN layer is very low, rest of the polymer electrolyte remains in solid-state. The cation (Na^+^) and the anion (CF_3_SO_3_^−^) both must contribute to capacitive action.

Important performance parameters of the optimised device are summarized in Table 1.

**Table 1:** Electrochemical performance parameters for devices fabricated in different configurations for a discharge current of 1 mA at 2 V operating potential. The (*) represents SSC performance close to an average value

Composition	*C*_s_ (F g^−1^)	*C*_ar_ (mF cm^−2^)	*E*_s_ (W h kg^−1^)	*P*_s_ (W kg^−1^)	ESR (Ω)	Efficiency
Electrode (1000 m^2^ g^−1^) without solvent layer	175* (160–185)	68* (60–70)	14* (12–17)	641* (610–650)	230* (180–250)	100* (90–100)
Electrode (1000 m^2^ g^−1^) with solvent layer	215* (200–225)	84* (80–90)	21* (18–23)	708* (680–720)	95* (80–120)	100* (90–100)
Electrode (1800 m^2^ g^−1^) with solvent layer	263* (240–275)	102* (95–105)	32* (30–35)	787* (770–800)	54* (40–70)	100* (90–100)

Finally, the supercapacitors developed in the present work are also compared with previously investigated Na^+^ ion-based supercapacitors. Evidently, the AN-incorporated SSCs exhibit comparable performance. In fact, it exhibits superior capacitance retention and coulombic efficiency. The present results are comparable to those of many supercapacitors based on gel/liquid electrolytes, as shown in Table 2.

**Table 2:** Performance parameters as reported previously on Na^+^/other ion-based supercapacitors for a comparison with the present investigation. RT stands for room temperature

S. no	Electrolyte	Electrolyte type	Electrode	Operating temperature	*σ* (Ω^−1^ cm^−1^)	*E*_s_	*P*_s_	*C*_s_	ESR	Coulombic efficiency	Capacitance retention (operating potential)	Ref.
1	10NaI–90[PEO_1−*x*_NZSP_*x*_]	Solid	Activated carbon (∼877 m^2^ g^−1^)	30 °C	∼10^−4^	∼44 W h kg^−1^	1.9 kW kg^−1^	104 F g^−1^	878 Ω	70%	∼20% after 400 cycles (at 2 V)	22
2	10NaCF_3_SO_3_–90(0.40PEO–0.60NZSP)	Solid	Activated carbon (1000 m^2^ g^−1^) activated carbon	40 °C	∼10^−4^	∼3.55 W h kg^−1^	∼607 W kg^−1^	∼150 F g^−1^	∼50 Ω	∼100%	∼93% after 2500 cycles (at 1 V)	5
3	3 M KOH^+^ 0.1 M K_3_[Fe(CN)_6_]	Liquid	CoFeO_4_|AC	RT	Liquid like	50.34 W h kg^−1^	1450 W kg^−1^	693 F g^−1^	—	100%	∼91% after 5000 cycles	36
5	PVdF-HFP/IL/DPA/KI	Gel	Porous activated carbon	RT	4.52 × 10^−3^	73.2 W h kg^−1^	34.8 kW kg^−1^	337 F g^−1^	20–25 Ω cm^2^	98–100%	74% after 6000 cycles (at 2.5 V)	37
6	PC/PMMA/LiClO_4_	Gel	P[E-CNDTT-E] films	RT	Gel-type	50 W h kg^−1^		223 F g^−1^		100%	90% after 8000 cycles	38
7	PVA–K_2_SO_4_	Gel	Vanadium disulfide–black phosphorus	RT	Gel	29 μW h cm^−2^	597 mW cm^−2^	203.3 mF cm^−2^	20 Ω	∼100%	87% after 10 000 cycles (at 1 V)	39
8	Carbon	Solid	10NaCF_3_SO_3_–90(0.40PEO–0.60NZSP)	50 °C	3 × 10^−4^	32 W h kg^−1^	780 W kg^−1^	∼260 F g^−1^	∼50 Ω	∼100%	90% after 10 000 cycles (1 V)	Present work

## 4. Conclusions

The present work may be summarized below:

(1) It is demonstrated that the performance of solid-state Na^+^ ion-based supercapacitors can be notably enhanced by introducing an organic polar solvent at the electrode–electrolyte (solid–solid) interface. This solvent stays stable and helps in interfacial charge movement. The amount of this layer is very low and preserves the solid-state nature of the electrolyte membrane.

(2) A gel-like layer forms on the surface of the CSPE when AN is added. This layer helps establish better interfacial contacts and is stable even with repeated cycling. The results also suggest that the charge movement across the interface becomes smooth, faster, and reversible to a reasonable extent. It helped improve specific capacitance substantially.

(3) The capacitance retention after approximately 10 000 cycles is about 60% at 2 V and 1 mA, and 90% at 1 V and 1 mA. These devices maintain high coulombic efficiency and reasonably low ESR. The same can be further improved at lower operating voltages. Also, the device shows appreciable stability at high discharge currents (5 mA) and operating potentials up to ∼3 V.

(4) Equivalent Series Resistance (ESR) is the sum of different resistive contributions: electrolyte, electrode–electrolyte interface, active material, and current collector. We have demonstrated that the solvent layer substantially modifies the interfacial resistance by providing smooth contact, thus reducing ESR. To further reduce ESR, the thickness of the polymer electrolyte should be decreased. Additionally, the choice of binder and electrode material conductivity can affect ESR and should be investigated.

(5) Utilizing the surface area of the activated material in solid-state supercapacitors has been challenging. This study offers a solution to this issue. After AN addition, the surface area of the activated carbon plays a vital role in device performance. Specific capacitance approaches to ∼260 F g^−1^ from ∼210 F g^−1^ as the surface area of active material increases from ∼1000 m^2^ g^−1^ to ∼1800 m^2^ g^−1^. Other device parameters have also shown significant improvement. The gel layer helps in utilizing the surface area effectively. Long cycling performance is improved notably after addition of AN.

(6) A Series combination of 5 supercapacitor cells could glow an 8 V LED circuit for more than ∼30 min, readily confirming the potential of these cells in practical applications.

(7) The authors believe that such a solvent layer approach may also be attempted in solid-state pseudo supercapacitors where the diffusion and charge transfer are limited due to the solid–solid interface. Various low evaporation rate solvents should be attempted to see their effect on surface modification.

## Data availability

All relevant data are available from the corresponding authors upon reasonable request.

## Conflicts of interest

There are no conflicts to declare.

## Supplementary Material

RA-015-D4RA08292C-s001
