# Supplementary material for: Achieving high-performance parameters in NASICON-polymer composite electrolyte-based solid-state supercapacitors by interface modification
Source: RSC Adv. 2025 Feb 27;15(9):6518–30. doi: 10.1039/d4ra08292c (PMC11865907; doi:10.1039/d4ra08292c)
Supplement: RA-015-D4RA08292C-s001 [file RA-015-D4RA08292C-s001.pdf]

Supplementary Information (SI) for RSC Advances.
This journal is © The Royal Society of Chemistry 2025

Supplementary Document

Achieving high-performance parameters in NASICON-polymer composite electrolyte-based solid-state supercapacitors by interface modification

Neha, Anshuman Dalvi*

Department of Physics, BITS Pilani-Pilani Campus (RJ-333031), India

*Corresponding Author email: adalvi@pilani.bits-pilani.ac.in

Table S1. Fitted Data parameters of Figure 6c.

**Sample\Parameter**	**R_b (Ω)**	**R_{ct} (Ω)**	**ESR(Ω)**
00_AN	111	6	118
01_AN	65	9	74
03_AN	35	7	44
05_AN	33	4	37

Table S2. Pore volume, diameter, and surface area of the activated carbon as obtained from BET analysis (Figure 7a).

**Parameter/ Surface Area**	**1000m^2-g^{-1}**	**1500m^2-g^{-1}**	**1800m^2-g^{-1}**
Total pore volume (p/p0=0.9877) [cm^3g^{-1}]	0.5585	0.698	0.766
Average pore diameter (nm)	1.7746	1.8764	2.112

Table S3. Fitted Data parameters of Figure 7b

**Sample\Parameter**	**R_b (Ω)**	**R_{ct} (Ω)**	**ESR (Ω)**
1000 m^2-g^{-1}	37	27	66
1500 m^2-g^{-1}	16	17	48
1800 m^2-g^{-1}	14	6	24
